# The Impact of Incarceration on Obesity: Are Prisoners with Chronic Diseases Becoming Overweight and Obese during Their Confinement?

**DOI:** 10.1155/2015/532468

**Published:** 2015-03-18

**Authors:** Madison L. Gates, Robert K. Bradford

**Affiliations:** ^1^Institute of Public and Preventive Health, Georgia Regents University, 1120 15th Street, CJ-2300, Augusta, GA 30912, USA; ^2^Georgia Correctional HealthCare, Georgia Regents University, 1499 Walton Way, HS 3507, Augusta, GA 30912, USA

## Abstract

*Introduction*. The association between incarceration and weight gain, along with the public health impact of former prisoners who are overweight or obese, warrants more investigation to understand the impact of prison life. Studies regarding incarceration's impact on obesity are too few to support assertions that prisons contribute to obesity and comorbid conditions. This study examined a statewide prison population over several years to determine weight gain. *Methods*. Objective data for weight, height, and chronic diseases, along with demographics, were extracted from an electronic health record. These data were analyzed statistically to determine changes over time and between groups. *Results*. As a total population, prisoners not only gained weight, but also reflected the distribution of BMIs for the state. There were differences within the population. Male prisoners gained significantly less weight than females. The population with chronic diseases gained less weight than the population without comorbid conditions. Prisoners with diabetes lost weight while hypertension's impact was negligible. *Conclusion*. This study found that weight gain was a problem specifically to females. However, this prison system appears to be providing effective chronic disease management, particularly for prisoners with diabetes and hypertension. Additional research is needed to understand the impact incarceration has on the female population.

## 1. Introduction

Obesity is a pandemic that is impacting health and healthcare costs of populations around the globe [1–3]. Prisoners, often referred to as offenders in the United States, belong to a population that spans the globe and share social, environmental, and health characteristics associated with the obesity pandemic [4, 5]. For example, many offenders, regardless of the country where they are incarcerated, have the following characteristics: low socioeconomic status, limited access to healthcare prior to incarceration, substance use disorders, and greater probability of having infectious and chronic diseases [4, 5]. Studies have discussed similar health issues and problems with the provision of care in all types of correctional systems, including high and low income countries, as well as a range of countries defined by different systems of government; differences often pertain more to magnitude and extent instead of type of problems [4, 5]. However, there are variations in the state and quality of healthcare that correctional systems provide.

A few studies have suggested that the social and structural environment of prisons contribute to obesity, exacerbate chronic diseases, and are an obstacle for offenders to either maintain or improve their health [4, 6]. These studies and the state of correctional health around the globe are the reasons why public health professionals, researchers, and educators should have an interest in corrections and offenders. The health risks for individuals who are overweight or obese are clear, in that, these individuals are at much greater risk of developing conditions, such as hypertension, type 2 diabetes, coronary heart disease (CHD), and stroke, as well as mental health problems, such as depression, compared to their normal weight peers [6, 7].

Studies have proposed that the design of correctional facilities themselves, which control offenders' freedom of movement and options for caloric intake, is a contributor to weight gain [4–6]. For example, caloric intake in corrections seldom includes fresh fruits, vegetables, or low fat and low sodium options; security concerns regarding offender and staff safety necessitate restricting and controlling movement [5, 6]. Despite these potential contributors to obesity, there is a paucity of correctional health research investigating weight gain, despite the impact on public health (i.e., most offenders return to their communities when they complete their sentences).

The health of offenders has an impact on public health when they are reintegrated into the general population, which occurs on a daily basis [8]. Unmanaged obesity and risk factors, such as hypertension, heart problems, and diabetes in corrections ultimately impact public health resources and communities to which offenders return. The offender population should be considered as a vulnerable population, since the vast majority of those incarcerated offenders have had limited access to a healthcare system, largely due to limited or no financial resources and inadequate or no health insurance [9–13]. The limited or no access to healthcare has resulted in many offenders having a poor health history when they became incarcerated.

National correctional health data from 2004 indicated that a large percent of offenders had hypertension (13.8%), heart problems (6.1%), and diabetes (4.0%) and these medical conditions were often compounded by the fact that many offenders had a history of alcohol abuse (44.6%) and drug dependency (44.3%) [14]. Also, many offenders prior to incarceration had limited access to health care, engaged in unhealthy behaviors, and had high rates of chronic and infectious diseases [14]. Limited access to healthcare, low health literacy, and unhealthy behaviors contribute to offenders being a vulnerable population; as a group, they experience health disparities (poorer health outcomes and greater incidence of diseases compared to other populations) in terms of mental and behavioral health, substance use disorders, infectious diseases, and chronic diseases.

The aim of this study was to contribute to our understanding of incarceration and obesity and to investigate the impact that corrections have on offenders, particularly the population that has comorbid diseases, such as hypertension, hyperlipidemia, and diabetes.

## 2. Methods

### 2.1. Procedures

This retrospective longitudinal study for 2005–2011 was approved by an institutional review board at an academic health center and conducted with a statewide department of corrections (DOC) in the east south central region of the United States. Data for this study were extracted from a statewide department of corrections' electronic health record (EHR) and offender management system (OMS), which contains demographic and nonhealth related information, such as type of offense committed, parole date, and education level.

The EHR that the DOC uses is a complete health record and includes physical, mental, and dental health information, as well as pharmacy, laboratory, and vital statistics. Health information, such as diagnoses and vital statistics, was entered into the system by health care professionals and data, such as pharmacy and laboratory values, and were transmitted to the EHR from an external source. All OMS data were entered into the system by correctional officers. The population for this DOC is comprised of male and female offenders who reside in facilities located throughout the state. All offenders have an OMS record and an EHR, which remain active until the offender dies or is released back into the community. All offenders with an active EHR between 2005 and 2011, two or more valid observations for weight, height, and incarceration duration greater than zero were included in the study.

### 2.2. Analysis

Diagnoses of hypertension, type 2 diabetes, and hyperlipidemia and risk factors for obesity and being overweight were extracted and linked to the patient's pharmacy record to determine who had active prescriptions. The beginning weight and height and last recorded weight during the study period were extracted and used to calculate the beginning and ending body mass index (BMI). See Table 1 for a complete list of variables collected.

BMI was calculated as weight in kilograms divided by height in meters squared (bmi = weight  (kg)/height  (m)^2^). This study used the Centers for Disease Control and Prevention (CDC) ranges for BMI, as shown in Table 2.

The rate of change in BMI (ΔBMI) was calculated as the difference in BMI divided by the beginning BMI. This measure (ΔBMI) was calculated to evaluate whether or not offenders gain weight during their incarceration (a primary aim of this study). The rate of change in BMI measures the rate in which BMI changes, which allows for comparison across BMI ranges; for example, a 1.0 rate of change in BMI can be detected and compared to those who are underweight, as well as offenders who are obese. Duration of incarceration was calculated using the date of incarceration and the end date for the study.

Using SAS 9.2, differences in beginning and ending weight and BMI were examined using paired* t*-test to determine whether or not ending weight minus beginning weight were significantly different from zero. The one sample* t*-test was used to investigate differences in ΔBMI. Correlations between BMI, ΔBMI, age, and incarceration duration also were conducted to determine what relationships, if any, existed between variables. To make group comparisons, such as race, the nonparametric Kruskal-Wallis rank sum test was used to examine whether or not variance between groups was equal.

## 3. Results

The population for this statewide department of corrections (DOC) in 2011 was 10841 offenders.  Table 3 describes the population for the DOC in which the majority of offenders were male, white, had a twelfth grade education, had a primary offense of larceny, and were classified as medium level security. However, 50% or more of the observations for education level, primary offense, and security level were missing.

Observations that could not be paired for beginning weight, date for beginning weight, ending weight, and date for ending weight were excluded from the study, as were the observations that had an incarceration duration equal to zero. As a result of the exclusions, there were 2932 valid observations. There were 2715 (93%) males and 217 (7%) females, and the race distribution was comprised of 1903 (65%) whites, 949 (32%) African Americans, and 32 (1%) of all other races, which represented the actual distribution of race for the DOC in 2011. The percent of female offenders was less than the actual 9.9%. The large percent of missing data for education level, primary offense, and security level precluded any descriptive or inferential analyses with these variables. Hypertension (630, 21%) emerged as the most prevalent chronic disease followed by dyslipidemia (499, 17%) and diabetes (142, 5%).

### 3.1. Population Changes in Weight

The mean age for offenders included in this study was 40, which was greater than the actual mean age of 38 for males and 37 for females, and the mean length of incarceration was 2 years (See Table 4). Table 4 shows that offenders entered corrections overweight and that there was a modest increase in ending BMI. Offenders also had a positive rate of change in BMI (ΔBMI) during their incarceration.

The mean weight change for the population was an increase of 0.96 kg and the mean change in BMI was 0.15, as shown in Table 5. In other words, offenders gained weight during their incarceration.

**Figure 1:**
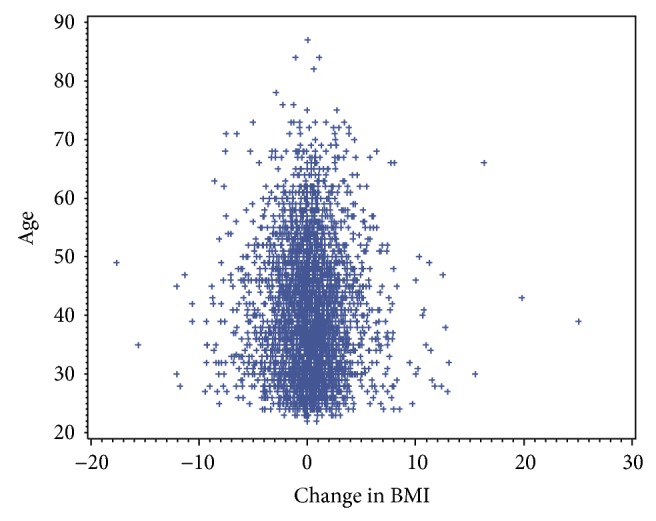
Age distribution for change in BMI.

The one sample* t*-test revealed significant population differences for the ΔBMI, which was used to standardize weight change. The one sample* t*-test indicated that the mean Δ BMI was 1.02, which was significantly different from zero, *t*(2931) = 5.9275, *P* = 0.000, and 95% CI [0.68,1.35]. Gender and hypertension were the only variables where there was a significant change in BMI (*P* < 0.001). Diabetes (*P* = 0.058) was slightly greater than *P* = 0.05 level of significance, but there were no other significant differences to include hyperlipidemia, race, length of incarceration, and age. Interestingly, age was not meaningfully correlated with BMI (See Figure 1) or ΔBMI; older offenders were neither more overweight/obese than younger offenders nor were they gaining more weight.

### 3.2. Population Differences for Changes in Weight

**Figure 2:**
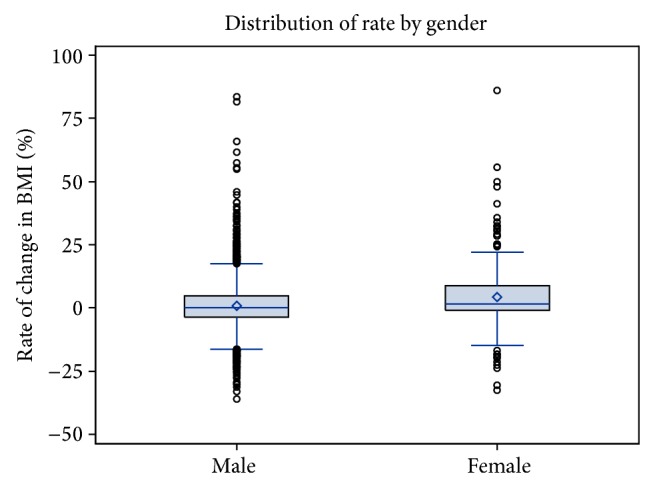
Rate of change in BMI during incarceration.

Further analyses of gender revealed that female offenders had a significantly greater rate of change in BMI (ΔBMI) during incarceration than males. The mean ΔBMI for female offenders was 5.34 (CI [4.63, 6.05]) during their incarceration compared to 0.67 (CI [0.65, 0.69]) for males, as shown in Figure 2. The ΔBMI for female offenders was 7.97 times that of males.

The results of this study indicated that offenders (males and females) gained weight during incarceration; female offenders gained significantly more weight than males. However, offenders with diabetes (ΔBMI = −0.39) and hypertension (ΔBMI = 0.06) did not gain more weight than offenders who did not have these chronic diseases.

## 4. Discussion

This study found that offenders gained weight and increased their BMI during incarceration, as other studies have indicated. Surprisingly, chronic diseases, such as diabetes and hypertension, were not explanatory for individuals who were overweight or obese. Race also was not a factor, despite the fact that African Americans adults have the highest rate of obesity compared to other groups, such as Mexican Americans and whites [15]. Despite the many nonsignificant findings, gender was highly relevant; female offenders gained more weight than their male counterparts.

In a meta-analysis, Herbert et al. [5] found obesity to be prevalent among female offenders. Herbert et al. [5] evaluated multiple offender studies throughout the world and found differences between offenders and nonoffenders and disparities between high and low income countries. Male and female offenders were more likely to be overweight or obese compared to their nonoffender counterparts and high income countries had a higher prevalence of obesity than low income countries [5].

Clarke and Waring [16] conducted a study in a unified jail and prison for women and found that 35% of the population was overweight and 32% were obese. During a median of 2-week period, 71% of offenders experienced weight gain during their incarceration. In fact, offenders had a mean weight gain of 0.50 kg per week (SD = 0.95 kg, 95% CI [−1.50, 4.17]) [16]. However, offenders who were incarcerated 2 weeks or less had greater weight gain (0.77 kg) compared to women with longer periods of incarceration (0.37 kg) [16]. Shorter durations resulted in about 2.2 times more weight gain than longer durations [16]. However, this study found no relationship between being overweight or obese and length of incarceration; that is, there were no differences between recently incarcerated offenders and offenders who have been incarcerated for several years.

This study, unlike others, was conducted institution-wide for a 7-year period and found that female offenders were more likely to gain weight and to be overweight or obese compared to male offenders, which raised a number of issues for correctional health, such as the impact that imprisonment may be having on female offenders. There have been a few reports and studies that have described or explored the differences in services and programs provided to female offenders [17–20]. The suggestion is that female offenders are provided inadequate services, programs (e.g., work release), and recreational activities compared to male counterparts and that these differences have an adverse effect on their physical and mental health [17–20]. The weight gain disparity between males and females for this statewide department of corrections (DOC) may only be limitedly explained by the opportunities men have for work release and recreational and physical activities compared to women. Similar to other correctional systems, this DOC had significantly fewer female offenders than males and as such had smaller facilities and fewer programs; in other words, female offenders have more sedentary lifestyles.

Herbert et al. [5] proposed that high income countries made no distinction between the energy intake provided to males and females, even though females require less. The energy intake issue was compounded by the fact that the most high income countries provided foods that exceeded dietary recommendations for sodium and fat [5]. Along with energy intake, atypical antipsychotic medications may be explanatory for the differences in weight gain between female and male offenders. Atypical antipsychotic medications have been associated with weight gain, because they can disrupt metabolic regulation [21–23]. The female population for this DOC utilized mental health services and was prescribed more atypical antipsychotic medications than the male population.

The gender differences for mental health problems in this DOC are consistent with the national data. Glaze and James [24] reported that 73% of female offenders in prison had mental health problems compared to 55% of males and the number of offenders who are prescribed medication increased from 12% to 15% during the same time period [24]. However, we do not suggest that a mental health diagnosis alone contributes to obesity and there is no evidence that all antipsychotic medications are associated with weight gain.

The finding that chronic diseases were not associated with weight gain for offenders (male and female) was surprising. The rate of change in BMI (ΔBMI) for offenders with diabetes or hypertension was significantly different from the ΔBMI for offenders who did not have these diseases. In fact, offenders with diabetes and a prescribed medication had a mean ΔBMI of −0.39 compared to nondiabetics at 1.09. The finding for offenders with hypertension who were prescribed antihypertensive medication was similar, in that offenders who not diagnosed with hypertension had a ΔBMI of 1.28 compared to a 0.06 for offenders with hypertension. Initially, these findings appeared counterintuitive. Yet, there are explanatory reasons why there is a “positive” health disparity in favor of patients with chronic diseases.

Unlike primary care clinics for the nonincarcerated population, correctional health has more access to information about its patients than the general public and has been able to overcome many of the obstacles that complicate care for the nonincarcerated population. There are no transportation issues (primary care for this DOC is conducted in the prison).

1. Correctional clinics have a robust reminder system to minimize missed appointments.
2. Correctional health is a managed care organization and has more or less eliminated issues regarding access to primary care, as mandated by the 1976 U.S. Supreme Court Estelle v. Gamble decision.
3. There is a strong continuity of care, since patients, in effect, belong to one health care practice, which shares and has access to the same health information.

Correctional institutions also provide what they call chronic care clinics. When an offender is diagnosed with a chronic disease, the patient is assigned to the appropriate clinic and scheduled for recurrent visits based on national standards.

Offenders with chronic diseases typically are seen more often by a primary care provider than offenders who do not have chronic diseases, which may be explanatory for the weight gain disparity between those with and without diabetes and hypertension. In other words, their primary care provider has more opportunities to intervene in the offender's health, such as placing offenders on special diets. Offenders who do not belong to a chronic care clinic may only be seen by a primary care provider once a year for an annual physical. The relationship between weight gain and becoming obese or overweight and having a chronic disease may be an indicator of the success primary care providers in corrections have with chronic diseases and the availability of health data, such as vital statistics and lab values, accessed and managed via an electronic health record (EHR).

This study did not find a statistical difference in BMI or ΔBMI between African Americans and whites, which does not coincide with the national obesity data. In 2009-2010, Flegal et al. [15] in their estimate of the prevalence for obesity among adults found that African Americans had the highest age adjusted rates of obesity compared to Hispanics and whites. African American and Mexican American women had greater increases in prevalence of obesity during the 10-year period that ended in 2010 than other populations [15]. With respect to race, this study found no weight gain disparities, despite the overrepresentation of African Americans as offenders and the greater prevalence of diabetes and hypertension.

The data that emerged from this study indicated that chronic diseases were not explanatory or a factor for the prevalence of obesity or weight gain among offenders. In fact, correctional health may have a management model that can be exported or adapted to the general population with respect to managing weight gain and preventing the onset or minimizing the effect of chronic diseases and excess weight gain.

## 5. Conclusion

Energy intake, programs, and atypical antipsychotic medication may be explanatory for the disparities between female and male offenders; however, there are no indicators that female offenders consume all the food they are provided. In addition to institutionally provided foods, correctional facilities typically have commissaries (markets) where offenders may purchase goods via credit they have earned from working in a correctional facility or funds they have received from an outside source, such as family members or friends. Goods from the commissaries include food items, many of which are processed high sodium and high fat content foods.

Food purchases from the commissary also are only an approximation of what offenders consume instead of, or in addition to, their institutional meals. Offenders sometimes engage in proxy purchases for other offenders or trade commissary goods as a form of currency. This study did not collect data regarding offenders who were on special diets, what they purchased from the commissary, what specific medications offenders were prescribed, or whether or not they were taking atypical antipsychotic medications. However, atypical antipsychotic medications may be a factor related to the significant difference in weight gain between female and male offenders.

This study found health disparities within a statewide department of corrections where female offenders are gaining significantly more weight than males. Other correctional systems around the globe may also have significant population differences regarding health outcomes and statuses and this study has discussed potential contributors, as well as positive findings, related to obesity that may be broadly applicable to correctional systems and that transcend geography and geopolitics.

Future studies will benefit from understanding the relationship between obesity and social factors, such as educational level, jobs that offenders perform during their incarceration (a potential indicator of physical activity), security level (e.g., minimum, medium, and maximum), primary offense, and outcome data, such as HbA1c, blood pressure, lipid panels, and degree of disease control. Along with social factors and health outcome data, interviews with a diverse sample of offenders and former offenders, particularly female offenders, may be helpful to learn what psychosocial issues may be barriers to effectively addressing obesity and weight gain. Understanding the impact these factors have on excess weight gain will be instrumental in developing an intervention for offenders and changing policy, especially regarding female offenders, who are at greater risk of becoming overweight or obese than their male counterparts.

## Figures and Tables

**Table 1:** Variables.

Age	
Beginning weight	
Category of primary offense	
Chronic disease: ICD-9 code	
Diabetes: 250	
Hyperlipidemia: 272.4	
Hypertension: 401, 401.1, 401.9, and 997.91	
Date of incarceration	
Education level	
Ending weight	
Gender	
Height	
Race	
Security level	

**Table 2:** CDC BMI ranges for adults.

BMI	Weight status
Underweight	<18.5
Normal	18.5–24.9
Overweight	25.0–29.9
Obese	≥30.0

**Table 3:** Population demographics.

	Males	Females	Total
Race			
African American	3009	183	3192
Asian	10	1	11
Latino	146	4	150
Native American	11	1	12
Pacific Islander	3	1	4
White	6298	877	7175
Unknown	290	7	297
Total	**9767**	**1074**	**10841**
Education level			
Primary school	51	4	55
Middle school	440	52	492
Less than high school	1425	242	1667
Twelfth grade	1728	141	1869
Some college	294	57	351
2-year degree	51	13	64
Bachelor degree	27	5	32
Graduate degree	9	2	11
Doctoral degree	3		3
Subtotal	**4028**	**516**	**4544**
Missing	5739	558	6297
Category of primary offense			
Drugs	380	35	415
Homicide	765	73	838
Larceny	928	34	962
Nonviolent	196	26	222
Sex	703	11	714
Violent	337	18	355
Subtotal	**3309**	**197**	**3506**
Missing	6458	877	7335
Security level			
Community (level 1)	130	32	162
Minimum (level 2)	318	25	343
Medium (level 3)	2284	127	2411
Close (level 4)	621	22	643
Maximum (level 5)	124	2	126
Subtotal	**3477**	**208**	**3685**
Missing	6290	866	7156
Total	**9767**	**1074**	**10841**

**Table 4:** BMI changes during incarceration (total population).

	Min	Max	Median	Mean
Age	22.0	87.0	38.0	39.8
Beginning weight (kg)	48.0	176.0	83.5	85.6
Ending weight (kg)	46.3	155.1	83.9	86.0
Height (m)	1.2	2.1	1.8	1.8
Beginning BMI	15.0	60.5	26.5	27.4
Ending BMI	15.7	56.4	26.8	27.5
ΔBMI (%)	−33.2	86.1	0.7	1.0
Duration (days)	2.9	2935	784.6	752.6

**Table 5:** Paired sample *t*-test: weight and BMI.

	Mean	C95 confidence interval		*t*	df	Sig.
		Lower	Upper			
Weight	0.96	0.32	1.60	2.95	2931	0.002
BMI	0.15	0.06	0.25	3.22	2931	0.000
